# *Campylobacter* species prevalence, characterisation of antimicrobial resistance and analysis of whole-genome sequence of isolates from livestock and humans, Latvia, 2008 to 2016

**DOI:** 10.2807/1560-7917.ES.2019.24.31.1800357

**Published:** 2019-08-01

**Authors:** Irēna Meistere, Juris Ķibilds, Lāsma Eglīte, Laura Alksne, Jeļena Avsejenko, Alla Cibrovska, Svetlana Makarova, Madara Streikiša, Lelde Grantiņa-Ieviņa, Aivars Bērziņš

**Affiliations:** 1 Institute of Food Safety, Animal Health and Environment BIOR, Riga, Latvia

**Keywords:** Campylobacter, C.jejuni, C.coli, typing, antimicrobial resistance, Latvia, food-borne infections, zoonotic infections, surveillance, whole genome sequencing

## Abstract

**Background:**

*Campylobacter* is the main cause of bacterial gastroenteritis worldwide. The main transmission route is through consumption of food contaminated with *Campylobacter* species or contact with infected animals. In Latvia, the prevalence of campylobacteriosis is reported to be low (4.6 cases per 100,000 population in 2016).

**Aim:**

To determine prevalence, species spectrum and antimicrobial resistance (AMR) of *Campylobacter* spp. in Latvia, using data from various livestock and human clinical samples.

**Methods:**

We analysed data of *Campylobacter* microbiological monitoring and AMR (2008 and 2014–16) in Latvia. Data from broilers, poultry, pigs, calves and humans were used to determine prevalence of *Campylobacter*. Additionally, 45 different origin isolates (22 human) were sequenced on the Illumina MiSeq platform; for each isolate core genome multilocus sequence typing was used and relevant antimicrobial resistance mechanisms were identified.

**Results:**

Overall, *Campylobacter* prevalence in was 83.3% in pigs, 50.2% in broilers, 16.1% in calves and 5.3% in humans; *C. jejuni* was the predominant species in all sources except pigs where *C. coli* was main species. High level of resistance in *Campylobacter* were observed against fluoroquinolones, tetracycline and streptomycin, in most of sequenced isolates genetic determinants of relevant AMR profiles were identified.

**Conclusions:**

In Latvia, prevalence of *Campylobacter* in livestock is high, especially in pigs and broilers; prevalence in poultry and humans were lower than in other European countries. AMR analysis reveals increase of streptomycin and tetracycline resistant broiler origin *C. jejuni* strains. WGS demonstrates a high compliance between resistance phenotype and genotype for quinolones and tetracyclines.

## Introduction

The most common cause of bacterial gastroenteritis is campylobacteriosis, which is an infection caused by *Campylobacter* spp. bacteria. More than 200,000 cases were reported in 2016 in Europe [1]. Latvia has one of the lowest rates of campylobacteriosis in the European Union (EU) with 4.6 cases per 100,000 inhabitants, however, it is probable that the majority of infections are not reported. For comparison, the prevalence of *Campylobacter* in other Baltic countries is much higher, with 22.6 and 42.4 cases per 100,000 inhabitants in Estonia and Lithuania, respectively [1].

The main reservoirs of *Campylobacter* spp. are birds, cattle and pigs as the bacteria are a part of their enteric microflora. The main source of human infection is contaminated fresh meat, particularly poultry (due to its high consumption) and milk products [2]. There is also evidence that infection can be acquired from contaminated water [3] or mud [4].

A previous study in Latvia focused on the prevalence of *Campylobacter* in broiler production (samples collected from chickens in slaughterhouses, hereafter called broilers) and retail level (poultry), with 92.5% and 56.3% prevalence in pooled intestine and carcasses samples, respectively [5]. Detailed research of antimicrobial resistance (AMR) showed that a high proportion of *Campylobacter* strains in broilers originating from Latvia were resistant to fluoroquinolones (100% to ciprofloxacin and 87.9% to nalidixic acid) and streptomycin (39.6%) [6].

Whole genome sequencing (WGS) has been demonstrated as a suitable method for routine surveillance and outbreak investigation of various infectious diseases including campylobacteriosis [7-9] and could be a powerful and reliable tool in AMR monitoring and phenotype prediction [10,11].

The aim of this study was to analyse the prevalence, species spectrum and AMR of *Campylobacter* spp. in various livestock and in human clinical samples. WGS analysis was used to identify the mechanisms of AMR and to evaluate the genetic heterogeneity and potential transmission routes between sources.

## Methods

### Sample collection

Animal and food samples were collected and tested within the Latvian National Monitoring Programme for Campylobacter according to European Union (EU) Directive 2003/99/EC [12], EU AMR monitoring according to Implementing Decision 2013/652/EU [13] and the Latvian National Research programme. In 2008, sequential samples of broiler skin and caeca were collected. In 2014 and 2016, sequential caeca samples were collected from two main broiler factories in Latvia. Each caeca sample was pooled from 10 individual birds. The skin and caeca sample groups did not differ significantly (chi-squared test, p = 0.462) in 2008, resulting in one merged group. The poultry samples were acquired as a part of the regular monitoring programme from retail stores in 2016.

Pig caeca samples were collected from 40 slaughterhouses covering all regions of Latvia during 2015, each sample was pooled from five individual animals.

Faecal specimens from healthy calves were collected from individual animals aged less than 1 year from 19 dairy cattle farms covering all territory of Latvia in 2015.

Routinely tested human stool samples from sporadic cases of gastroenteritis were analysed for gastrointestinal bacterial pathogens. The samples were received 2015–16 from two regional acute care hospitals, each covering a population of ca 50,000. The isolated *Campylobacter* spp. strains were further analysed according to the AMR monitoring programme.

### Ethical statement

Ethical approval was not required as human samples were routinely collected and patients’ data remained anonymous. The planning conduct and reporting of study was in line with the Declaration of Helsinki, as revised in 2013.

### Isolation and detection of *Campylobacter* spp.

The animal/food and human stool samples were tested in separate laboratories. All animal and food samples were cultured according to ISO 10272 for the detection of thermophilic *Campylobacter* on mCCDA agar (Biolife, Milan, Italy) under microaerobic conditions at 41.5 °C for 44 hours. Human stool samples were cultured according to in-house method on mCCDA agar (Biolife) under microaerobic conditions at 41.5 °C for 44 hours. Suspicious colonies were cultivated on Columbia blood agar and analysed after 24–44 hours. Identification of cultures was based on colony morphology, microscopic appearance (e.g. motility) and phenotypic characteristics, including the production of catalase and oxidase, hydrolysis of hippurate and indoxylacetate, followed by multiplex PCR [14] or mass spectrometry (matrix-assisted laser desorption/ionisation time-of-flight, Bruker Daltonics Biotyper). *Campylobacter* isolates were stored in Brain heart infusion broth (Biolife) with 20% glycerol at - 80 °C until further investigation.

### In vitro antimicrobial susceptibility testing

*C. jejuni* and *C. coli* isolates were tested for antimicrobial susceptibility by microdilution method in cation-adjusted Mueller-Hinton broth with 5% lysed horse blood (TREK Diagnostic Systems Ltd. East Grinstead, United Kingdom) and minimal inhibitory concentration (MIC) panel (Sensititre, reference EUCAMP2, TREK diagnostic Systems Ltd.).

The susceptibility of *Campylobacter* isolates was determined following the standard M45-A2 for fastidious bacteria form the Clinical and Laboratory Standards Institute [15]. According to European Committee on Antimicrobial Susceptibility Testing (EUCAST) guidelines epidemiological cut-off values (ECOFFs) were set: nalidixic acid 16 µg/mL, ciprofloxacin 0.5 µg/mL, gentamycin 2 µg/mL, streptomycin 4 µg/mL, erythromycin 4 µg/mL and 8 µg/mL, tetracycline 1 µg/mL and 2 µg/mL for *C. jejuni* and *C. coli*, respectively [16]. An isolate was defined as multidrug-resistant (MDR) when resistance to three or more nonrelated antimicrobials was observed.

### Genomic DNA extraction and whole genome sequencing

A group of isolates was selected for WGS analysis including 23 human origin *Campylobacter* spp. (22 *C. jejuni* and one *C. coli*), supplemented by 22 isolates from other sources with the most relevant AMR profile [17]. Prior to genomic DNA extraction, the bacteria were cultured for 24h at 41.5 °C on Columbia broth agar with 5% lysed horse blood (TREK Diagnostic Systems Ltd.). Enzymatic lysis was used for DNA isolation and purification was done on silica-membrane, both according to QIAamp DNA Mini Kit protocol (QIAGEN Manchester Ltd. Manchester, United Kingdom).

Sequencing libraries were constructed using 1 ng DNA with Nextera XT Library preparation kit (Illumina, San Diego, California (CA), United States (US)) and sequencing was performed on Illumina Miseq platform, generating 2x300-bp paired-end reads according to the manufacturer’s instructions. First, quality trimming for each sample was done until the average Phred quality was 30 in a window of 20 bp. Trimming was followed by de novo genome assembly using Velvet version 1.1.04 [18]. The expected genome size was 1.64 Mb. The quality of genome assembly was assessed by N50 (minimum 10,000 bp accepted) and the mean genome coverage (minimum 30x) that ranged from 39x to 117x, with 87x on average. All samples with quality parameters below these settings were re-sequenced.

### Data analysis

Subsequently assembled genomes were analysed by the Ridom SeqSphere ^+^ 5.0.0 software (Ridom, Muenster, Germany) [19]. For genome characterisation gene by gene comparison of *Campylobacter* *jejuni/coli* multilocus sequence type (ST) seven loci and core genome (cg)MLST 637 loci were used. The newly identified alleles were submitted to the cgMLST nomenclature server (www.cgmlst.org) maintained by Ridom. The minimum spanning tree based on cgMLST 637 loci, pairwise ignoring missing values, was generated to evaluate the relationships between all *Campylobacter* spp. isolates and to identify potential outbreaks (cluster-alert distance of 13 cgMLST targets was set as the maximum absolute distance of different cgMLST targets used to indicate the epidemiological relationship between two samples (www.ridom.de). For selected isolates, kSNP3.0 software was used for single nucleotide polymorphism (SNP) identification and reference-free phylogeny analysis [20].

The genes and mutations encoding mechanisms of AMR were surveyed in assembled contigs with the Resistance Gene Identifier (RGI) 4.0.1 and The Comprehensive Antibiotic Resistance Database (CARD) 2.0.0 [21]. The applied cut-off values were 85% for sequence identity and 50% for sequence length (cut-offs were not applied in mutation screening).

The presence of virulence factors was determined by screening assembled contigs against virulence factor database of virulence genes, version 18 March 2018 [22] with ABRicate (https://github.com/tseemann/abricate), using the default settings. For gene presence determination, the cut-off values were 85% for sequence identity and 50% for sequence length. For some genes, for example, *flaA* and *flaB*, the length cut-off was set at 20% due to these genes often being disrupted by gaps between contigs.

The chi-squared test and Fischer’s exact test were used where applicable to calculate the odds ratios (OR) and 95% confidence intervals (CI) by using two-by-two frequency tables of the respective overlaps and considering p values of < 0.05 statistically significant.

### Sequence data deposition

All raw reads generated were submitted to the European Nt Archive (http://www.ebi.ac.uk/ena/) under the study accession number PRJEB26725.

## Results

### Prevalence of *Campylobacter* spp. in livestock and human samples

Between 2008 and 2016, the presence of *Campylobacter* spp. was tested in a total of 1,303 samples, with 434 (33.3%) found to be positive. The prevalence of *Campylobacter* spp. was significantly different across sources (Table 1; p values reported in Supplement S1).

**Table 1:** *Campylobacter* species prevalence in various sources, Latvia, 2008–2016 (n = 1,303)

Source	Year	Total samples tested	Negative		Positive		*Campylobacter jejuni*		*Campylobacter coli*		*Campylobacter lanienae*	
			n	(%)	n	(%)	*n*	(%)	n	(%)	n	(%)
**Broilers**	2008	271	151	55.7	120	44.3	109	90.8	11	11 9.2	0	0 0.0
**Broilers**	2014	147	54	36.7	93	63.3	93	100.0	0	0.0	0	0.0
**Broilers**	2016	90	48	53.33	42	46.7	42	100.0	0	0.0	0	0.0
**Poultry**	2016	31	27	87.1	4	12.9	4	100.0	0	0.0	0	0.0
**Calves**	2015	180	151	83.9	29	16.1	24	82.8	5	17.2	0	0.0
**Pigs**	2015	150	25	16.7	125	83.3	2	1.6	114^a^	91.2	11^a^	8.8
**Humans**	2015–16	434	415	95.6	23	5.3	22	95.7	1	4.3	0	0.0
Total		**1,303**	**869**	**66.7**	**434**	**33.3**	**292**	**67.3**	**131**	**30.3**	**11**	**2.5%**

^a^ Samples contained *Campylobacter* *coli* and *Campylobacter lanienae*.

For samples from broilers, 255 (50.6%) were positive for *Campylobacter* (Table 1). *C. jejuni* was the dominant bacterial population and was detected in 95.7% of all *Campylobacter* isolates while *C. coli* was found only in some samples in 2008.

Poultry samples from retail stores had significantly lower prevalence when compared with broilers from slaughterhouses: 12.9% vs 3 years pooled prevalence 50.6% (OR: 0.15; 95% CI: 0.05–0. 43; p 0.00005) (Supplement S1) and only *C. jejuni* was detected.

The highest *Campylobacter* prevalence (83.3%) was observed in pigs caeca, two of 127 isolates contained *C. jejuni*, 114 (91.2%) contained *C. coli* and 11 (8.8%) *C. lanienae*. Of 11 cases positive for *C. lanienea*, two were also positive for *C. coli*. *C. lanienae* was detected exclusively in pigs caeca samples.

Of 180 calf faecal samples, 29 were *Campylobacter* positive and the predominant species was *C. jejuni* (82.8%).

Human faecal samples had the lowest *Campylobacter* prevalence (5.3%). Of 23 positive cases, 22 contained *C. jejuni*.

### Antimicrobial resistance and multidrug resistance in different *Campylobacter* spp. isolates

**Figure 1:**
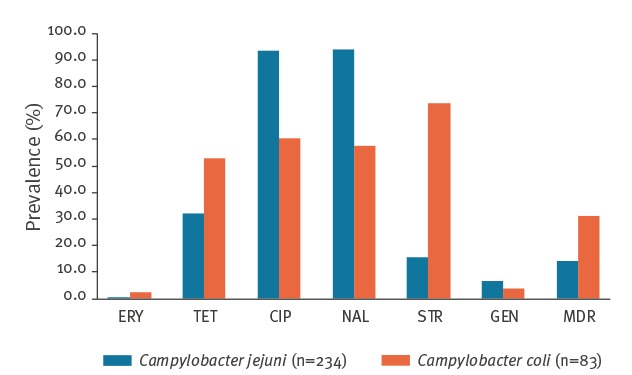
Prevalence of resistant isolates of *Campylobacter* *jejuni* and *Campylobacter* *coli*, isolated from broilers, poultry, calves, pigs and humans, Latvia, 2008–2016 (n = 317)

CIP: ciprofloxacin; ERY: erythromycin; GEN: gentamycin; MDR: multidrug-resistant; NAL: nalidixic acid; STR: streptomycin; TET: tetracycline.

When combining the isolates from all sources (i.e. broilers, poultry, calves, pigs and humans), there were 434 *Campylobacter* spp. isolates, 317 of which (234 *C. jejuni* and 83 *C. coli*) were analysed for antimicrobial susceptibility. *C. Jejuni* showed the highest resistance against ciprofloxacin (93.6 %) and nalidixic acid (94 %). *C. coli* was most resistant against streptomycin (73.5%), which was higher than observed for *C. jejuni* (15.8%). Both species showed a low resistance against gentamicin and erythromycin, with 6.4% and 0.4% in *C. jejuni* and 3.6% and 2.4% in *C. coli*, respectively (Figure 1).

The data from broilers were compared between years 2008, 2014 and 2016 and resistance of *C. jejuni* against tetracycline increased significantly from 14.0% in 2008 to 42.5% in 2016 (OR: 0.22; 95% CI: 0.08–0.59; p 0.0022) and resistance against streptomycin increased from 14.0% in 2008 to 45.0% in 2016 (OR: 0.20; 95% CI: 0.08–0.53; p 0.001) (Table 2), accordingly MDR isolates increased from 12.5% in 2008 to 42.5% in 2016 (OR: 0.19; 95% CI: 0.07–0.52; p 0.0009), however in all 3 years the total number of analysed *Campylobacter* isolates were less than 100 and larger sample set should be analysed to confirm our results.

**Table 2:** Antimicrobial resistance of *Campylobacter* species by origin

Source and year of sampling	Species	Number of isolates tested	Number of isolates (resistant/total)						
			ERY	TET	CIP	NAL	STR	GEN	MDR
Broilers, 2008	*C. jejuni*	57	0/57	8/57	57/57	57/57	8/57	10/57	7/57
	*C. coli*	8	0/8	1/8	8/8	8/8	2/8	1/8	1/8
Broilers, 2014	*C. jejuni*	93	1/93	22/93	93/93	93/93	1/93	3/93	0/93
Broilers, 2016	*C. jejuni*	40	0/40	17/40	39/40	39/40	18/40	0/40	18/40
Poultry, 2016	*C. jejuni*	4	0/4	3/4	3/4	3/4	2/4	0/4	2/4
Pigs, 2015	*C. jejuni*	1	0/1	1/1	1/1	1/1	1/1	0/1	1/1
	*C. coli*	71	1/71	39/71	38/71	36/71	55/71	1/71	21/71
Calves, 2015	*C. jejuni*	21	0/21	14/21	13/21	14/21	4/21	2/21	2/21
	*C. coli*	3	0/3	3/3	3/3	3/3	3/3	1/3	3/3
Humans, 2015	*C. jejuni*	18	0/18	10/18	13/18	13/18	3/18	0/18	3/18
	*C. coli*	1	1/1	1/1	1/1	1/1	1/1	0/1	1/1

C: *Campylobacter;* CIP: ciprofloxacin; ERY: erythromycin; GEN: gentamycin; MDR: multidrug-resistant; NAL: nalidixic acid; STR: streptomycin; TET: tetracycline.

The occurrence of MDR in other origin *C. jejuni* isolates had lower prevalence of MDR, e.g. 9.5% for calf faecal samples and 16.7% for human clinical isolates. For *C. coli* 29.6% of pig origin, the only human origin and three of three isolates showed MDR (Table 2).

### Typing of *Campylobacter* spp. isolates

Livestock and food isolates of *C. jejuni* were assigned to six different clonal complexes (CCs) representing nine known sequence types (ST) (Table 3). CC-383 included two broiler isolates with ST-5 and two poultry isolates with ST-6461. Additional poultry sample belonged to CC-283/ST-383. All *C. jejuni* isolates from calves belonged to CC-21 and were identified as ST-21 and ST-806. The only pig origin C. jejuni analysed belonged to the same type as broilers CC-383/ST-5. From eight analysed *C. coli* four pig origin and two calf origin isolates showed previously unknown ST. Only two of pig origin *C. coli* isolates represented previously known CC-828/ST-828 and ST-854.

**Table 3:** Sequence diversity of human and livestock samples, by sequence clonal complex, multilocus sequence type and core genome multilocus sequence type, Latvia, 2008–2016 (n = 45)

Clonal complex	Multi locus sequence type	Core genome multi locus sequence type	Origin (isolates with certain genotype)
21	19	1542	Human (1)
		1598	Human (1)
	21	1544	Calf (2)
		1545	Calf (2)
	50	587	Human (1)
		1541	Human (1)
		1547	Human (2)
	806	1595	Calf (2)
	1519	205	Human (1)
206	572	435	Human (1)
		1596	Human (1)
283	267	193	Human (1)
	383	251	Poultry (1)
353	5	1546	Broiler (2), Pig (1)
	6461	338	Human (3), Poultry (2)
443	51	1543	Human (4), Pig (1)
464	464	1592	Human (1)
48	918	1597	Calf (1)
49	49	1593	Human (3)
828	828	1563	Pig (1)
	854	1539	Pig (1)
	8328	1591	Human (1)
ND	ND	1528	Human (1)
		1529	Calf (2)
		1535	Pig (1)
		1537	Pig (1)
		1538	Pig (1)
		1540	Pig (1)

ND: not determined.

Human isolates were assigned to eight CCs represented by 10 multilocus STs. The predominant CC belonged to ST-50 (4/23), ST-19 (2/23) and ST-1519 (1/23). In other CC groups, only one ST per group was represented: CC-443 in four isolates with ST-51; CC-353 in three isolates with ST-6461; and CC-49 in three isolates with ST-49. The *C. coli* isolate did not belong to any known sequence group.

**Figure 2:**
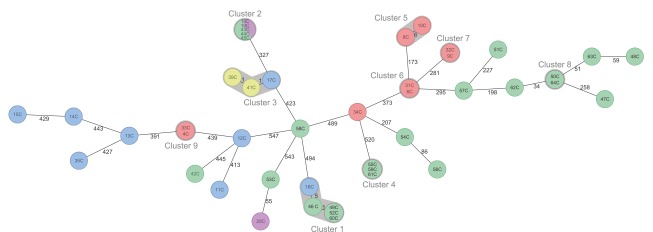
Minimum spanning tree of 45 *Campylobacter* isolates

A comparison of human origin *Campylobacter* genomes based on gene by gene typing of *Campylobacter* *jejuni/coli* core genome scheme was also conducted. Four different cgMLST clusters (Figure 2; numbered 1, 2, 4 and 8) of human isolates were identified where more than one case representing the same or closely related cgMLST. Ten human isolates represented sporadic cases and were therefore unrelated to the other samples. The available dates of sample collection (Supplement S2) were used to confirm the possible link between genetically related cases. Cluster 1 included three identical human isolates, one-allele distant isolate and one pig origin isolate only five alleles distant, indicating a possible source of infection. Cluster 2 included five isolates in total – three human clinical samples and two poultry meat samples. Although the human samples were collected during 2015 and food samples in 2016, zero allele distance was observed using cgMLST gene by gene typing. The following reference-free SNP comparison showed less than nine different nt positions between isolates (data not shown). In two cases isolates from patients related by place and time were clustered together (clusters 4 and 8).

C: *Campylobacter;* cgMLST: core genome multilocus sequence typing.

Distance based on 637 columns from *C. jejuni/coli* cgMLST, pairwise ignoring missing values. *C. jejuni/coli* cgMLST Complex Type/Cluster-Alert distance: 13. The lengths of the edges are not proportional to the numbers. Colours represent the following sample origin: green: human; red: calf; blue: pig; yellow: broiler; purple: poultry; grey background indicates genetically related isolates. Four different clusters (1;2;4;8) with human isolates involved were identified.

### Identification of antimicrobial resistance determinants

Antimicrobial resistance determinants were examined using the whole genome sequences and compared with known phenotype data (Table 4 and Supplement S2). In total, 30 *Campylobacter* isolates showed resistance to quinolones and all of them contained a point mutation in the *gyrA* gene coding for DNA gyrase subunit A (Thr-86-Ile). The second most common type of resistance was against tetracyclines and all resistant isolates harboured a *tet(O)* gene. Resistance against aminoglycosides arose from the *ant(6)-1b* gene that was found in 9 of 12 isolates displaying high resistance against streptomycin (i.e. > 16 µg/mL). In three cases, an additional *aph(3’)-IIIa* gene encoding aminoglycosides phosphotransferase was detected, suggesting that more than one simultaneous mechanism can give rise to resistance against antimicrobial drugs. Two streptomycin-resistant isolates demonstrated gentamicin resistance as well, in one case the isolate contained *ant(6)-1b* and *aph(3’)-IIIa* genes, but in another case the mechanism of resistance was not identified. Additionally, in all of the sequenced isolates, multidrug and bile efflux pump coding operon *cmeABC* was detected (Supplement S2).

**Table 4:** Comparison of *Campylobacter* species resistance and corresponding mechanisms in whole genome sequence, Latvia, 2008–2016

Antimicrobial drug	Drug class	Resistance mechanism, gene or mutation	Resistance principle	Isolates with resistant phenotype	Isolates with resistance mechanism
Nalidixic Acid	Quinolones	*gyrA* Thr-86-Ile	Antibiotic target alteration	30	30
Ciprofloxacin		*gyrA* Thr-86-Ile	Antibiotic target alteration	30	30
Tetracycline	Tetracyclines	*tet(O)*	Antibiotic target protection	27	27
Streptomycin	Aminoglycosides	*ant(6)-Ib*	Antibiotic inactivation	12	9
		*APH(3')-IIIa*	Antibiotic inactivation	12	3
Gentamycin		*ant(6)-Ib*	Antibiotic inactivation	2	0
		*APH(3')-IIIa*	Antibiotic inactivation	2	0
Erythromycin	Macrolides	23S RNA mutations	Antibiotic target alteration	1	0

### Virulence-associated genes

All genomes were examined for a broad range of virulence-associated genes, including motility, adherence, invasion and toxin genes; the majority of genes were represented in all of the analysed genomes, except for a few cases (Supplement S2). Some of the analysed virulence genes were present only in *C. jejuni*, but not in *C. coli*: N-linked glycosylation gene (*pglG)*, cytochrome C551 peroxidase (*cj0020c)* and major antigenic peptide (*peb3)*. Three genes i.e. *cdtA*, *cdtB,* and *cdtC* (coding for the cytolethal distending toxin (CDT) subunits A, B and C, respectively) were confirmed in all *C. jejuni* isolates; in *C. coli*, only genes *cdtB* and *cdtC* were detectable. In a single *C. jejuni* isolate from pigs caeca, several genes of the virulence plasmid pVir was identified. This plasmid encodes the type IV secretion system and could be potentially involved in adhesion and invasion. Of note, the *wlaN* gene associated with Guillain–Barré syndrome was identified in six *C. jejuni* isolated from calves’ faeces and in seven of 23 human origin *C. jejuni* isolates, all the *wlaN* carriers belonged to CC-21.

## Discussion

In this study, the prevalence of *Campylobacter* spp. in various sources were analysed focussing on AMR and the respective gene diversity, resistance determinants and virulence factors. The highest prevalence was observed in pigs where 83.3% analysed samples contained *Campylobacter*, mainly *C. coli*, followed by broilers with average 50.3% positive samples, mainly *C. jejuni*.

Although the prevalence of *Campylobacter* observed in livestock in Latvia was higher than on average in the EU, the proportion of positive cases in fresh broiler meat was lower than the EU average in 2016, according to data from 14 countries [1]. Broiler meat is considered as one of the major sources of campylobacteriosis and lower contamination rate can partly explain the low number of human campylobacteriosis cases in Latvia.

The broiler population in Latvia has been studied before and more than 90% of broilers intestine samples contained *Campylobacter* in 2010 [5]. Here, we report data collected from the same two broiler slaughterhouses in 2008, 2014 and 2016 where we found the lowest prevalence in 2008 and the highest in 2014, suggesting that there is not an overall tendency but various factors can affect *Campylobacter* contamination in the broiler flocks. In addition, Kovalenko et al. reported *C. coli* in samples from the investigated slaughterhouses in 2010 [6]; we observed *C. coli* in 2008, but not in 2014 and 2016. These data can serve as the starting point for deeper investigation of broiler population to evaluate the factors contributing to the dynamics of *Campylobacter* infection.

Monitoring of AMR of zoonotic bacteria has revealed an alarming trend of *C. jejuni* resistance profile in broilers. The proportion of streptomycin and tetracycline resistant isolates have significantly increased when comparing data from 2008 and 2016. Latvia has one of the highest proportions of fluoroquinolone-resistant *C. jejuni*, at 97.5% compared with the average proportion in the EU at 67%. Tetracycline, erythromycin and gentamycin resistance was at similar level for Latvia and the EU [23].

In all of the quinolone-resistant isolates, mutation in the DNA gyrase subunit A, resulting in Thr-86-Ile substitution, was identified and this was not found in any of the sequenced susceptible isolates. This mutation has been previously linked to the quinolone-resistance together with less common mutations Asp-90-Asn, Ala-70-Thr in the same region have been reported [24], but the latter were not detected in selected set of isolates. The *Campylobacter* determinants of resistance against aminoglycosides were quite diverse. The streptomycin-resistant (MIC > 16 mg/mL) strains in most cases carried *ant(6)-Ib* gene, however for two isolates no previously published mechanisms could be attributed. For another *C. jejuni* isolate showing low level resistance (MIC < 4mg/mL) against both streptomycin and gentamycin, we did not find any known aminoglycoside-specific resistance mechanism. Three *C. coli* isolates from various hosts showed resistance to streptomycin and in one case also to gentamycin and contained the *aphA(3’)-IIIa* gene addition to *ant(6)-Ib* gene. No phenotypic differences were observed when comparing isolates with one or both resistance genes. In 2017, Cantero et al. performed WGS on 16 *C. jejuni* and *C. coli* strains isolated from broilers and did not find molecular mechanisms responsible for streptomycin resistance in most of the strains. The presence of undiscovered genes, possibly encoded in plasmids, was assumed [25].

One of the most interesting findings in our study was the identification of *bla-OXA-184* gene in five isolates from human clinical samples and a pig sample. Analysis by cgMLST demonstrated these isolates clustered together, pointing to a link between human and pig strains. In 34 other isolates from various hosts, the *bla-OXA-61* gene was identified, which has been previously reported as present in *C. jejuni* and *C. coli* [11,26]. Since the routine AMR monitoring for *Campylobacter* spp. does not include the beta-lactam class of antibiotics, the genotype to phenotype accordance cannot be determined in this case. In all of the sequenced isolates three genes of MDR efflux pump cmeABC were detected. CmeABC is an intrinsic resistance mechanism against fluoroquinolones, macrolides and cephalosporines in *Campylobacter* spp. and is constitutively expressed in wild type strains [27]. Since this mechanism was found in all strains independently of their resistance profile, additional mechanisms responsible for clinically significant level of related drug resistance must be identified and deeper analysis looking for genetic variation related to resistance level should be performed.

WGS data analysis was also used to characterise the diversity of *Campylobacter* populations from various sources and to study the correlation between sources and genotype. The *C. jejuni* population in calves was mainly characterised by the presence of CC-21. The same CC was isolated from dairy cattle in Lithuania [28] and Austria [29] and is widely distributed between humans and poultry [30]. CC-21 was the most common CC between human clinical isolates as well, but STs between human clinical isolates and calf samples do not overlap and calves or dairy products as infection source should not be assumed. CC-21 was represented in human clinical isolates and poultry samples from Lithuania and Estonia [28,31]; one ST was the same as isolated in from humans in Latvia (ST-50). In order to identify and confirm the possible source of this significant fraction of human isolates in Latvia, it is necessary to study a representative sample set of CC-21 including strains from various sources and countries, taking onto account the high rate of zoonotic transmission between different species [30].

Although only few *Campylobacter* strains from broilers and poultry could be included in WGS analysis, two interesting observations were made. First, we classified two broiler *C. jejuni* isolates as ST-5, the same type isolated in Estonia from poultry originating from Latvia [31]. The evidence from WGS can be used to confirm that systematic contamination happened in the food production chain. Second, we identified ST-6461 in human clinical isolates and poultry samples. All five isolates belonged to one and the same cgMLST, with no distant alleles, even though 18 months have passed between the collection of the first and last sample (the individual sample collection date reported in Supplement S2). We believe these human patients were infected from poultry and the genetic and other factors that have enabled the conservation of this genotype for prolonged periods of time should be further investigated.

To our knowledge, this is the first study in Latvia that aims to characterise *Campylobacter* prevalence from sources other than broilers and poultry, which up until now has been the main risk studied for campylobacteriosis in humans. In order to get a complete picture regarding the situation in Latvia, it is necessary to include a representative sample set in the analysis of WGS with special attention to broiler isolates, including those from neighbouring countries. In addition to significant data about the prevalence of certain genotypes, WGS data analysis can help to elucidate the genetic, virulence and resistance mechanisms. Characterisation of bacterial phenotypes and analysing these data complimentary to bacterial genetic profiles holds a great promise for deciphering the mechanisms of resistance [10,32]. Our findings demonstrate the importance of rigorous surveillance for *Campylobacter* contamination within the food manufacturing chains, using standardised analytical procedures and regular data sharing between at least the neighbouring countries.

## Supplementary Data

346000 Supplement S1

## Supplementary Data

55000 Supplement S2
