# Supplementary material for: Campylobacter species prevalence, characterisation of antimicrobial resistance and analysis of whole-genome sequence of isolates from livestock and humans, Latvia, 2008 to 2016
Source: Euro Surveill. 2019 Aug 1;24(31):1800357. doi: 10.2807/1560-7917.ES.2019.24.31.1800357 (PMC6685098; doi:10.2807/1560-7917.ES.2019.24.31.1800357)
Supplement: Supplement S1 [file 1800357_MEISTERE_SupplementS1.pdf]

Supplement S1

This supplementary material is hosted by Eurosurveillance as supporting information alongside the article "Campylobacter spp. prevalence, characterisation of antimicrobial resistance and analysis of whole-genome sequence (WGS) of isolates from livestock and humans, Latvia, 2008 to 2016" on behalf of the authors who remain responsible for the accuracy and appropriateness of the content. The same standards for ethics, copyright, attributions and permissions as for the article apply. Eurosurveillance is not responsible for the maintenance of any links or email addresses provided therein.

Statistical analysis of differences in the prevalence of Campylobacter between various sources (p values are presented in the matrix)

	**Broilers, 2008**	**Broilers, caeca, 2014**	**Broilers, caeca, 2016**	**Broilers, pooled 2008, 2014, 2016**	**Poultry, 2016**	**Calves, faeces, 2015**	**Pigs, caeca, 2015**	**Human, faeces, 2015**
**Broilers, 2008**	-	0.000209	0.693319	0.132129	0.000768	0.000000	0.000000	0.000000
**Broilers, 2014**	0.000209	-	0.012255	0.006386	0.000002	0.000000	0.000026	0.000000
**Broilers, 2016**	0.693319	0.012255	-	0.568534	0.000838	0.000000	0.000000	0.000000
**Broilers, pooled 2008, 2014, 2016**	0.132129	0.006386	0.568534	-	0.000052	0.000000	0.000000	0.000000
**Poultry, 2016**	0.000768	0.000002	0.000838	0.000052	-	0.000000	0.000000	0.034439
**Calfs, 2015**	0.000000	0.000000	0.000000	0.000000	0.000000	-	0.000000	0.000001
**Pigs, 2015**	0.000000	0.000026	0.000000	0.000000	0.000000	0.000000	-	0.000000
**Humans, 2015**	0.000000	0.000000	0.000000	0.000000	0.034439	0.000001	0.000000	-
